# Drug Coated Balloon-Only Strategy in De Novo Lesions of Large Coronary Vessels

**DOI:** 10.1155/2019/6548696

**Published:** 2019-02-03

**Authors:** Mark Rosenberg, Matthias Waliszewski, Florian Krackhardt, Kenneth Chin, Wan Azman Wan Ahmad, Giuseppe Caramanno, Diego Milazzo, Amin Ariff Nuruddin, Houng Bang Liew, Oteh Maskon, Angela Bento, Jean-Christophe Macia, Norbert Frey

**Affiliations:** ^1^Klinikum Aschaffenburg-Alzenau, Medizinische Klinik 1, Aschaffenburg, Germany; ^2^Medical Scientific Affairs, B. Braun Melsungen AG, Berlin, Germany; ^3^Department of Cardiology and Internal Medicine, Charité–Universitätsmedizin Berlin, Campus Virchow, Berlin, Germany; ^4^Pantai Hospital, Kuala Lumpur, Malaysia; ^5^Pusat Perutban Universiti, Malaysia; ^6^Ospedale San Giovanni di Dio, Italy; ^7^The National Heart Institute of Malaysia, Malaysia; ^8^Hospital Queen Elizabeth II, Malaysia; ^9^Pusat Perutban Universiti Kebangsaan, Malaysia; ^10^Espirito Santo Evora, Portugal; ^11^CHU Montpellier, France; ^12^Innere Medizin III, Universitätsklinikum Schleswig-Holstein, Campus Kiel, Germany

## Abstract

**Objectives:**

We analyzed the efficacy of drug coated balloons (DCB) as a stand-alone-therapy in de novo lesions of large coronary arteries. DCBs seem to be an attractive alternative for the stent-free interventional treatment of de novo coronary artery disease (CAD). However, data regarding a DCB-only approach in de novo CAD are currently limited to vessels of small caliber.

**Methods:**

By means of propensity score (PS) matching 234 individuals with de novo CAD were identified with similar demographic characteristics. This patient population was stratified in a 1:1 fashion according to a reference vessel diameter cut-off of 2.75 mm in small and large vessel disease. The primary endpoint was the rate of clinically driven target lesion revascularization (TLR) at 9 months.

**Results:**

Patients with small vessel disease had an average reference diameter of 2.45 ± 0.23 mm, while the large vessel group averaged 3.16 ± 0.27 mm. Regarding 9-month major adverse cardiac event (MACE), 5.7% of the patients with small and 6.1% of the patients with large vessels had MACE (p=0.903). Analysis of the individual MACE components revealed a TLR rate of 3.8% in small and 1.0% in large vessels (p=0.200). Of note, no thrombotic events in the DCB treated coronary segments occurred in either group during the 9-month follow-up.

**Conclusions:**

Our data demonstrate for the first time that DCB-only PCI of de novo lesions in large coronary arteries (>2.75 mm) is safe and as effective. Interventional treatment for CAD without permanent or temporary scaffolding, demonstrated a similar efficacy for large and small vessels.

## 1. Introduction

Metallic stents are still the standard of care for percutaneous coronary interventions (PCI). However, such permanent metallic implants can lead to chronic inflammation within the vessel wall and induce neoatherosclerosis which may explain an ongoing risk for target lesion failure (TLF) after stent implantation [1]. Therefore, an interventional strategy to exclusively dilate coronary lesions without permanently caging the vessel seems to be very promising.

Drug Coated Balloons (DCB) were introduced for the delivery of antiproliferative drugs such as paclitaxel to the coronary vessel wall to inhibit neointimal proliferation after angioplasty. Nevertheless, there are only few data available which report the outcomes of the DCB-only strategy in previously untreated de novo coronary lesions. Our group and others have recently shown that a DCB-only approach as stand-alone therapy for de novo coronary artery disease (CAD) is associated with low rates of target lesion revascularization (TLR) and major adverse cardiac events (MACE) [2–5]. However, most of the cited studies were conducted in small coronary vessels with diameters of less than 2.75 mm. The recently published clinical endpoint trial BASKET SMALL [6] revealed that, in patients with de novo lesion of reference vessel diameter <3 mm, DCB angioplasty using the paclitaxel-iopromid matrix coating was noninferior to recent generation Drug Eluting Stents (DES) in terms of MACE (DCB 7.5% vs. DES 7.3%, p=0.918). Moreover, the meta-analysis by Megaly and coworkers [7] came to the same conclusion that DCB angioplasty in patients with small sized de novo lesions had similar TLR rates as those treated with DES.

Indeed, PCI in small vessels only accounts for approximately 35% of all coronary interventions [8]. Hence, knowledge of the safety and efficacy of such a DCB-only approach in de novo lesions of larger vessels is still insufficient.

We therefore used data from the international, multicenter DCB-only All-Comers Registry [2] to assess the potential of a DCB-only strategy according to the recommendations by the German Consensus Group [9] in large coronary vessels in de novo lesions.

## 2. Methods

### 2.1. Patient Population

To test our hypothesis we analyzed data from the international, multicenter DCB-only registry that prospectively enrolled patients in 8 European and 6 Malaysian centers [2]. For data presented in this manuscript we stratified patients according to their vessel diameter. Since there is no generally accepted definition, we chose a cut-off of 2.75 mm to distinguish between large and small coronary arteries [10, 11]. Lesion length was restricted to 25 mm. Moreover, we only considered patients that were treated with a DCB-only approach of de novo coronary lesions. Patients with additional in-stent restenosis (ISR) were included.

Since the DCB-only registry was designed as a real world evaluation of the paclitaxel-iopromid coated DCB, the only patient related exclusion criteria were contraindications for dual antiplatelet therapy (DAPT), nonavailability for clinical follow-up, or lack of informed consent. Based on national requirements in the participating countries (Germany, France, Italy, Portugal, and Malaysia), all mandatory authorizations were obtained from relevant ethics committees and/or government agencies. In France, this all-comers registry was approved by the Comité Consultatif sur le Traitement de l'Information en matière de Recherche dans le domaine de la Santé (CCTIRS) and the Commission Nationale de l'Informatique et des Libertés (CNOM). All patients gave written informed consent.

### 2.2. Study Procedure

A detailed description of the study procedure has been previously published [2]. Briefly, all investigators of the DCB-only registry were strongly endorsed to comply with the treatment recommendations of the German Consensus Group on how to use DCB's in CAD [9]. Predilatation was performed with uncoated balloons having a balloon-to-vessel ratio of 0.8-1.0. Subsequent DCB angioplasty was only conducted in the absence of a major, flow-limiting dissection, i.e., less severe that type C-F according to the NHLBI classification [12], and severe recoil. DCBs used in the study had a paclitaxel-iopromid matrix coating (SeQuent® Please, B. Braun Melsungen AG). The length of the DCB was chosen to exceed both lesion ends for at least 2 to 3 mm. As for the predilatation, the DCB diameters were adapted to the reference vessel diameters with a balloon-to-vessel ratio of 0.8-1.0. Recommended inflation time was at least 30 seconds at low dilatation pressures. Bail-out stent implantation in the case of severe dissections or residual coronary diameter stenosis of more than 30% was left to the discretion of the investigator.

Based on the all-comers approach, efforts were made to respect established clinical pathways regarding periprocedural medications. However, acetylsalicylic acid (ASA) 75-325 mg/d was recommended life-long and a clopidogrel loading dose of at least 300 mg, complemented with a regimen of 75 mg/d for 4 weeks, was recommended as the standard DAPT according to the DCB-only protocol. A minimum of 6 months of DAPT were recommended when additional stents were implanted. Prasugrel or ticagrelor could be used instead of clopidogrel based on applicable guidelines. Intravenous administration of heparin (70 IU/kg) or 5000 IU was recommended and supplemented when required. Preloading with clopidogrel, ticagrelor or prasugrel prior to the procedure was permissible.

### 2.3. Primary and Secondary Endpoints

As previously described [2], the clinically driven target lesion revascularization rate (TLR) at 9 months was the primary endpoint. Major adverse cardiac events (MACE) as a secondary endpoint were defined as the composite of TLR, cardiac death, myocardial infarction (MI), and definite vessel thrombosis also after 9 months of follow-up. MI had to be accompanied with typical clinical symptoms, relevant ECG changes, and/or elevated troponin T or troponin I increases (3x the upper limit of normal). To define acute/subacute vessel thrombosis the ARC criteria [13] were used. In case that the cause of death was unknown or undeterminable, mortality was defined to be of cardiac origin.

### 2.4. Data Collection

An established electronic data capture system [3, 4] was utilized to document all patient and lesion relevant data. National principal investigators in each country were responsible to assure the accuracy of their national datasets. Whenever plausibility checks revealed inaccuracies, source data verification were conducted in the affected centers.

### 2.5. Statistical Analysis

The Chi^2^-test or Fisher's exact test was used to detect differences between treatment groups when dichotomous variables were compared. Unpaired Student's t-test or the Whitney-Mann nonparametric test was applied for continuous variables. To check for normal distribution either the Kolmogoroff-Smirnoff or the Shapiro-Wilk tests was used. For all tests the significance level *α* was 0.05. Prior to propensity score (PS) matching a logistic regression analysis with MACE as the dependent variable was conducted on the entire data set. Using the nearest neighbor matching algorithm, PS matching was conducted with independent variables which had the most important predictive values. For all analyses SPSS version 24.0 (IBM, Munich, Germany) was used.

## 3. Results

### 3.1. Patient Population

**Figure 1:**
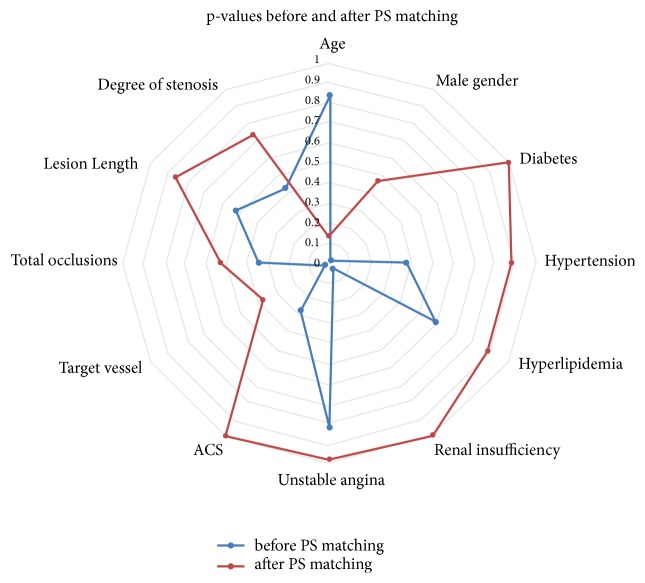
Demographic and lesion morphological characteristics before and after PS matching.

In the DCB-only registry 686 patients with de novo lesions were treated. Within this patient population 234 individuals were identified with similar cardiovascular and lesion morphological risk factors. While 117 of these patients revealed vessel sizes ≥2.75 mm, the other 117 examinees had angioplasty in coronary arteries with diameters <2.75 mm. Therefore, no significant differences between both groups regarding the most important cardiovascular risk factors were detected (Table 1). Of note, roughly one-third of the patients were diabetics or treated because of an acute coronary syndrome (ACS) (Table 1).  Figure 1 illustrates the p-values before and after PS matching of the most prominent predictors.

### 3.2. Lesion Morphology and Procedural Data

Targeted vessels were homogenously distributed between both treatment arms (Table 2). Due to the extensive cardiovascular risk profile rather complex coronary lesions were included in this registry. Enrolled patients had DCB-only angioplasty despite heavy calcifications, diffuse disease, chronic total occlusions, or aorto-ostial lesions. In both groups almost half of the patients revealed coronary stenosis classified as ACC/AHA type B2 or C (Table 2).

In the group of PS matched patients with DCB-only PCI in small vessels, reference vessel diameter averaged at 2.45 ± 0.23 mm, whereas patients with angioplasty in larger vessels had a reference vessel diameter of 3.16 ± 0.27 mm. Lesion lengths in both groups were comparable and calculated with 17.2 ± 9.8 mm and 17.4 ± 9.0 mm, respectively (p=0.856) (Table 2).

Predilatation rates in both treatment arms exceeded 90%. For subsequent DCB use in small vessels, 127 DCBs were used for 119 lesions. In large vessels DCB-only PCI was done with 132 DCB's for 126 lesions. Irrespective of group allocation DCB diameters matched the reference vessel diameter. Moreover, an average DCB length in both groups was selected that exceeded the lesion length by roughly 3 mm. In order to avoid overstretching, DCB inflation pressure was restricted to 9.3 ± 2.8 atm in small and to 9.7 ± 3.1 atm in larger vessels (p=0.434). Finally, DCB inflation time was longer than 50 seconds in both groups (Table 2).

Overall technical success was very high in both treatment arms. DCB-only angioplasty was successfully performed in all patients with small vessels and in 98.5% of patients with lesions in large coronaries (p=0.164). After DCB use, additional stents were implanted only in approximately 7% of the patients with small or large vessel lesions (p=0.880) (Table 2).

### 3.3. Clinical Follow-Up

All patients were available for in-hospital outcomes. Moreover, 106 of 117 patients (90.6%) with small vessel lesions underwent clinical follow-up after a mean of 8.5 ± 1.8 months, whereas 99 of 117 patients (84.6%) with DCB-only angioplasty in large vessels were available for follow-up after a mean of 8.8 ± 1.8 months (p=0.306) (Table 3).

In-hospital MACE rates were very low in both treatment arms and mainly triggered by postprocedural MI. However, overall event rates in both groups were too low to detect any differences regarding acute outcomes for DCB-only angioplasty in small or large vessel disease (Table 3).

At 9 months, we observed a MACE rate of 5.7% in small and 6.1% in large vessels (p=0.903). Hence, Kaplan-Meier Analysis for MACE did not indicate a significant difference between patients with small and large vessel disease as well (log-rank p=0.662). Analysis of the individual MACE components revealed a 9-month MI rate of 2.8% in small and 4.0% in larger coronary arteries. However, TLR rates after 9 months of 3.8% and 1.0% (Table 3), respectively, suggest that most of these ACS were caused by lesions which were not in the DCB-treated coronary segments. No patient died after DCB-only angioplasty in small vessels, while a 9-month cardiac death rate of 3.0% was noted in patients with larger vessels. The difference was not significant (p=0.071). Finally, no thrombotic events occurred after DCB-only angioplasty in small vessels, whereas in the patient cohort with larger arteries one definite vessel thrombosis was observed (p=0.300). Of note, the thrombosis was distal of the DCB-treated segment (Table 3).

## 4. Discussion

To the best of our knowledge we report for the first time the safety and efficacy data of a DCB-only approach according to the recommendations of the German Consensus Group [9] in large coronary arteries with previously untreated de novo lesions. Our main findings indicate that the use of a DCB-only strategy in large coronary arteries leads to similar results as compared to small vessel treatment, i.e., high technical success rates, low clinically driven TLR rates after 9 months, and a low conversion rate to bail-out stenting.

### 4.1. Lesion Preparation

These favorable results were achieved although almost half of the included coronary lesions were considered as complex. We believe that, in analogy to the overall registry, the low event rate in large coronary vessels is mostly explained by the strict compliance of all investigators to the treatment recommendations published by the German Consensus Group regarding the use of DCB in coronary artery disease. Key element in the use of a DCB-only strategy in de novo lesions is a proper lesion preparation by conventional balloon angioplasty. We observed a predilatation rate of almost 92% in large coronary arteries with a balloon-to-vessel ratio of 0.82. Furthermore, DCB devices were well matched according to the reference vessel diameter. Moreover, the DCB inflation pressure was rather low and long enough to secure gentle drug application to the adjacent vessel wall. Investigators were advised to proceed with additional stenting only in the case of flow limiting dissections (NHLBI class C-F) or high elastic recoil with residual stenosis exceeding 30% despite adequate predilatation. By following this interventional strategy, we were able to successfully use DCB's in 98.5% of the study population with large coronary vessels.

### 4.2. Bail-Out Stenting

Moreover, additional stenting and TLR rates after 9 months were as low as 3.8% (≤2.75 mm) and 1.0% (>2.75 mm), respectively. These outcomes compare favourably to other registries. For example, Wöhrle et al. [3] reported in the SeQuent Please World Wide registry an additional stenting rate of almost 19% in 559 patients treated with the DCB-only approach for de novo disease. While a DCB-only strategy without additional stenting resulted in a TLR rate of 1.0%, TLR rate increased to 2.4% if additional stents were implanted. Similar results were published by Waksman et al. in the Valentines II trial. In this study, 103 patients with de novo CAD were enrolled and treated with a DCB-only strategy. In comparison to our registry, the Valentines II investigator achieved a predilatation rate of 85% and reported an additional stent implantation in almost 12% of the patients. Hence, a TLR rate of only 2.9% after a 7.5-month follow-up was documented [5].

### 4.3. Clinical Results

We believe that the low TLR rates after DCB-only angioplasty in our patients with de novo lesions in large coronary arteries can be explained by the combination of proper lesion preparation, adequate DCB handling, and restrictive additional stent implantation.

On the other hand, our patient cohort with large vessels revealed a 9-month MI rate of 4%. Since TLR and TVR rates were 1% and 4%, respectively, we believe that most of the MIs during the follow-up period were spontaneous and not related to the previously DCB-treated coronary segments. Instead, our observed MI rate is rather a result of the ongoing risk for adverse ischemic events in a patient population with extensive cardiovascular risk factors. In addition, we report a 9-month cardiac death rate of 3% which is substantially higher compared to previously published studies. However, while other all-comers registries enrolled ACS patients in only roughly 25% of the cases, our study population consisted of 33% acute MI patients [3, 4, 14–16]. Depending on the type of MI and underlying individual risk factors the annual mortality after hospital admission for an acute MI still exceeds 10% [17–19]. We are therefore of the opinion that a 9-month cardiac death rate of 3% is an anticipated finding under the given clinical circumstances in our study population.

Previous studies demonstrated that the absolute late lumen loss after BMS implantation was very similar among various vessel sizes. These findings suggest that comparable neointimal proliferation in terms of thickness will lead to relatively higher rates of in-stent restenosis, target lesion revascularizations, and eventually MACE in smaller coronary arteries [20–22]. On top of this, any permanent or temporary coronary implant will further reduce the minimum lumen diameter of a coronary artery and thereby additionally diminishing its compensatory space. Hence, it can be assumed that outcomes after exclusive dilatation of coronary lesions without any coronary implants are less dependent on the vessel size. Based on these assumptions, we were indeed able to demonstrate that DCB-only PCI of lesions in coronary arteries with diameters exceeding 2.75 mm is associated with high technical success, low TLR, and stent conversion rates. Moreover, PS matching revealed similar outcomes for a DCB-only approach in large and small coronary arteries which supports our theory that outcomes after DCB-only PCI of previously untreated coronary lesions may be independent of the vessel diameter.

In summary our data show very promising results for DCB-only PCI of de novo lesions in large coronary arteries. Our data complements the literature of the DCB-only use in de novo lesions which is currently restricted to reports in small vessel disease. Finally our data underlines the idea that angioplasty of native coronary lesions without permanent or temporary implants is less dependent on vessel diameter than stent based PCI.

### 4.4. DAPT Duration

The paclitaxel-iopromid matrix DCB used in this trial is recommended with 4 weeks of DAPT in case that no additional stenting is indicated, provided that previously implanted DES mandate a longer DAPT. We conducted an additional analysis in the overall de novo DCB-only cohort and found that in lesions >2.75 mm the DAPT duration was 2.7±1.6 months whereas in lesions with reference diameters ≤2.75 mm, the DAPT duration was 2.8±1.6 months (p=0.583). Also we observed that around half of each subgroup had a recommendation for 4 weeks of DAPT (>2.75 mm: 53.3% vs. ≤2.75 mm: 48.1%, p=ns).

## 5. Conclusions

Our data demonstrate for the first time that DCB-only PCI in large coronary arteries with de novo lesions is equally as safe and as effective as in small vessel disease. We understand our results as a first indication that the idea of a long-term beneficial interventional treatment of de novo CAD without temporary or permanent coronary implants is also applicable to large coronary vessels. However, as mentioned above before broader recommendation can be given, these very promising results need further confirmation in randomized clinical trials.

## Figures and Tables

**Table 1:** Patient demographics.

	**Before PS matching**				**After PS matching**		
**Variable**	**de novo**	**≤ 2.75 mm**	**>2.75 mm**	**p-value**	**≤ 2.75 mm**	**>2.75 mm**	**p-value**
Number of patients	686	552	134	-	117	117	-

Number of lesions	731	577	154	-	127	132	-

Age (years)*∗∗*	63.5±11.2	63.5±11.1	63.4±11.8	0.836	65.5±11.2	63.3±11.3	0.132

Male gender	551 (80.3%)	433 (78.4)	118 (88.1%)	0.012	97 (82.9%)	101 (86.3%)	0.469

Diabetes	303 (44.2%)	256 (46.4%)	47 (35.1%)	0.018	42 (35.9%)	42 (35.9%)	1.000

Hypertension	517 (75.4%)	420 (76.1%)	97 (72.4%)	0.373	85 (72.6%)	84 (71.8%)	0.884

Hyperlipidemia	504 (73.5%)	408 (73.9%)	96 (71.6%)	0.593	85 (72.6%)	84 (71.8%)	0.884

History of smoking	275 (40.1%)	221 (40.0%)	54 (40.3%)	0.956	46 (39.3%)	46 (43.6%)	0.507

Renal insufficiency	18 (2.6%)	11 (2.0%)	7 (5.2%)	0.036	5 (4.3%)	5 (4.3%)	1.000

unstable angina	205 (29.9%)	166 (30.1%)	39 (29.1%)	0.826	33 (28.2%)	32 (27.4%)	0.984

Atrial fibrillation							
paroxysmal	39 (5.8%)	29 (5.3%)	10 (7.5%)	0.373	5 (4.3%)	9 (7.7%)	0.683
persistent	19 (2.8%)	16 (2.9%)	3 (2.2%)		4 (3.4%)	2 (1.7%)	
long standing,	2 (0.3%)	1 (0.2%)	1 (0.7%)		1 (0.9%)	1 (0.9%)	
persistent							
permanent	19 (2.8%)	13 (2.4%)	6 (4.5%)		4 (3.4%)	6 (5.1%)	
no AF	607 (88.5%)	493 (89.3%)	114 (85.1%)		103 (88.0%)	99 (84.6%)	

Acute coronary Syndrome (ACS)	199 (29.0%)	155 (28.1%)	44 (32.8%)	0.276	39 (33.3%)	39 (33.3%)	1.000

STEMI	90 (13.1%)	74 (13.4%)	16 (11.9%)	0.652	15 (12.8%)	15 (12.8%)	1.000

NSTEMI	109 (15.9%)	81 (14.7%)	28 (20.9%)	0.077	24 (20.5%)	24 (20.5%)	1.000

*∗∗*independent t-test, otherwise Chi^2^ or Fisher's Exact Test whenever applicable.

**Table 2:** Lesion characteristics, procedural data, and device characteristics.

	**Before PS matching**				**After PS matching**		
**Variable**	**de novo**	**≤ 2.75 mm**	**>2.75 mm**	**p-value**	**≤ 2.75 mm**	**>2.75 mm**	**p-value**
Number of lesions	731	577	154	-	119	126	-

Target vessel							
LAD	299 (40.9%)	233 (40.4%)	66 (42.9%)	0.025	44 (37.0%)	53 (42.1%)	0.373
CX	252 (34.5%)	212 (36.7%)	40 (26.0%)		46 (38.7%)	34 (27.0%)	
RCA	155 (21.2%)	114 (19.8%)	41 (26.6%)		24 (20.2%)	32 (25.4%)	
graft	8 (1.1%)	4 (0.7%)	4 (2.6%)		2 (1.7%)	4 (3.2%)	
unknown	17 (2.3%)	14 (2.4%)	3 (1.9%)		3 (2.5%)	3 (2.4%)	

Total occlusion	56 (7.7%)	47 (8.1%)	9 (5.8%)	0.340	9 (7.6%)	7 (5.6%)	0.525

Chronic total occlusion	23 (3.1%)	20 (3.5%)	3 (1.9%)	0.338	7 (5.9%)	3 (2.4%)	0.166

Thrombus burden	21 (2.9%)	14 (2.4%)	7 (4.5%)	0.162	4 (3.4%)	4 (3.2%)	0.934

Diffuse vessel disease	330 (45.1%)	270 (46.8%)	60 (39.0%)	0.083	51 (42.9%)	52 (41.3%)	0.801

Calcification	144 (19.7%)	111 (19.2%)	33 (21.4%)	0.544	21 (17.6%)	30 (23.8%)	0.235

Vein graft	7 (1.0%)	3 (0.5%)	4 (2.6%)	0.019	1 (0.8%)	3 (2.4%)	0.342

Ostial lesion	143 (19.6%)	109 (18.9%)	34 (22.1%)	0.376	24 (20.2%)	29 (23.0%)	0.588

Bifurcation lesion	123 (16.8%)	97 (16.8%)	26 (16.9%)	0.983	18 (15.1%)	23 (18.3%)	0.512

Severe tortuosity	58 (7.9%)	48 (8.3%	10 (6.5%)	0.456	9 (7.6%)	10 (7.9%)	0.913

AHA/ACC type B2/C lesion	340 (46.5%)	267 (46.3%)	73 (47.4%)	0.803	57 (47.9%)	59 (46.8%)	0.866

Number of diseased vessels							
single	275 (37.6%)	220 (38.1%)	55 (35.7%)	0.184	50 (42.0%)	44 (34.9%)	0.314
double	274 (37.5%)	222 (38.5%)	52 (33.8%)		41 (34.5%)	42 (33.3%)	
triple	182 (24.9%)	135 (23.4%)	47 (30.5%)		28 (23.5%)	40 (31.7%)	

Reference diameter (mm)*∗∗*	2.45±0.41	2.31±0.26	3.15±0.26	-	2.45±0.23	3.16±0.27	-

Lesion length*∗∗*	17.4±8.6	17.2±8.8	17.6±8.3	0.517	17.2±9.8	17.4±9.0	0.856

Degree of stenosis (%)*∗∗*	84.7±11.8	84.9±11.9	83.9±11.5	0.429	84.0±14.9	84.6±10.6	0.738

DCBs used	772	609	163	-	127	132	-

Predilatation	721 (93.4%)	571 (93.8%)	150 (92.0%)	0.428	120 (94.5%)	121 (91.7%)	0.372

Predilation device diameter (mm)	2.19±0.42	2.00±0.23	2.50±0.61	<0.001	2.15±0.32	2.59±0.50	-

Predilatation device length (mm)	14.6±4.1	14.5±4.0	14.8±4.2	0.370	14.7±4.1	14.7±4.4	0.919

Predilatation pressure (atm)	11.6±3.5	11.4±3.4	12.5±3.8	0.002	12.1±3.4	12.6±3.8	0.298

DCB diameter (mm)	2.47±0.42	2.31±0.28	3.04±0.34	0.002	2.45±0.26	3.05±0.33	-

DCB length (mm)	20.6±4.9	20.7±4.7	20.4±5.2	0.061	20.3±4.9	20.1±5.2	0.751

DCB inflation pressure (atm)	9.2±2.8	9.1±2.7	9.5±3.1	0.493	9.3±2.8	9.7±3.1	0.434

DCB inflation time (sec)	50.9±16.5	50.2±16.4	53.5±16.8	0.599	50.7±17.0	53.4±15.7	0.264

Additional stent per DCB	46 (6.0%)	33 (5.4%)	13 (8.0%)	0.221	9 (7.1%)	10 (7.6%)	0.880

Overall technical success per lesion	765 (99.1%)	604 (99.2%)	161 (98.8%)	0.627	127 (100.0%)	130 (98.5%)	0.164

Multivessel PCI, non-target lesions	269 (36.8%)	230 (37.8%)	57 (35.0%)	0.512	33 (26.0%)	48 (36.4%)	0.072

BVS used in non-target lesions	8 (1.0%)	7 (1.1%)	1 (0.6%)	0.548	0 (0.0%)	1 (0.8%)*∗*	0.326

*∗∗*independent t-test, otherwise Chi^2^ or Fisher's Exact Test whenever applicable.

**Table 3:** Clinical outcomes.

	**Before PS matching**				**After PS matching**		
**Variable**	**de novo**	**≤ 2.75 mm**	**>2.75 mm**	**p-value**	**≤ 2.75 mm**	**>2.75 mm**	**p-value**
Number of patients	686	552	134	-	117	117	-

Patients with clinical follow-up	604 (88.0%)	493 (89.3%)	111 (82.8%)	0.038	106 (90.6%)	99 (84.6%)	-

Follow-up time (months)*∗∗*	8.7±1.7	8.7±1.7	8.8±1.7	0.523	8.5±1.8	8.8±1.8	0.306

In hospital MACE	7 (1.2%)	4 (0.8%)	3 (2.7%)	0.093	1 (0.9%	3 (3.0%)	0.313

In hospital Re-PTCA	2 (0.3%)	1 (0.2%)	1 (0.9%)	0.247	0 (0.0%)	1 (0.9%)	0.316

In hospital CABG	0 (0.0%)	0 (0.0%)	0 (0.0%)	-	0 (0.0%)	0 (0.0%)	-

In hospital MI	5 (0.8%)	2 (0.4%)	3 (2.7%)	0.016	1 (0.9%)	3 (2.6%)	0.313

In hospital cardiac death	2 (0.3%)	1 (0.2%)	1 (0.9%)	0.247	0 (0.0%)	1 (0.9%)	0.316

Acute Thrombotic events							
acute (<24h)	1 (0.2%)	0 (0.0%)	1 (1.1%)	0.082	0 (0.0%)	0 (0.0%)	-
subacute (>24h)	1 (0.2%)	1 (0.2%)	0 (0.0%)		0 (0.0%)	0 (0.0%)	

9-month MACE	34 (5.6%)	27 (5.5%)	7 (6.3%)	0.732	6 (5.7%)	6 (6.1%)	0.903

9-month TLR	14 (2.3%)	12 (2.4%	2 (1.8%)	0.689	4 (3.8%)	1 (1.0%)	0.200

9-month Re-PTCA	10 (1.7%)	9 (1.8%)	1 (0.9%)	0.490	4 (3.8%)	1 (1.0%)	0.200

9-month CABG	5 (0.8%)	4 (0.8%)	1 (0.9%)	0.916	0 (0.0%)	0 (0.0%)	-

9-month MI	22 (3.6%)	17 (3.4%)	5 (4.5%)	0.592	3 (2.8%)	4 (4.0%)	0.624

9-month cardiac death	6 (1.0%)	3 (0.3%)	3 (2.7%)	0.044	0 (0.0%)	3 (3.0%)	0.071

9-month TVR	25 (4.1%)	20 (4.1%)	5 (4.5%)	0.831	8 (7.5%)	4 (4.0%)	0.285

9-month non-target vessel revascularization	14 (2.3%)	10 (2.0%)	4 (3.6%)	0.319	2 (1.9%)	3 (3.0%)	0.596

9-month vessel thrombosis	2 (0.3%)	1 (0.2%)	1 (0.9%)	0.247	0 (0.0%)	1 (1.0%)*∗*	0.300

*∗*DES thrombotic event distal of DCB lesion.

*∗∗*independent t-test, otherwise Chi^2^ or Fisher's Exact Test whenever applicable.

## Data Availability

The raw data used to support the findings of this study have not been made available for proprietary/regulatory reasons by the manufacturer of this study device.

## Additional Points

Limitations. Patients in each individual study center were not consecutively included. In fact, the attempt to follow a DCB-only strategy was left to the discretion of the investigator. Additionally, 12.4% of the analyzed patients were not available for the 9-month clinical follow-up. Hence, a selection bias cannot be excluded. We therefore believe that our promising hypothesis generating data need confirmation in adequately powered randomized trials.
